# Recent advances of organic long persistent luminescence: Design strategy and internal mechanism

**DOI:** 10.1002/smo.20240034

**Published:** 2024-09-11

**Authors:** Jie Yang, Zhijian Chen, Manman Fang, Zhen Li

**Affiliations:** ^1^ Institute of Molecular Aggregation Science Tianjin University Tianjin China; ^2^ Joint School of National University of Singapore and Tianjin University International Campus of Tianjin University Binhai New City Fuzhou China; ^3^ Department of Chemistry Hubei Key Lab on Organic and Polymeric Opto‐Electronic Materials Wuhan University Wuhan China; ^4^ The State Key Laboratory of Luminescent Materials and Devices South China University of Technology Guangzhou China

**Keywords:** design strategy, internal mechanism, long persistent luminescence, organic systems

## Abstract

Organic afterglow materials have drawn increasing attention for their great potential in practical applications. Until now, most of them just show the lifetimes in milliseconds or seconds, while the realization of long persistent luminescence (LPL) lasting for minutes or even hours is difficult. In 2017, Adachi and Kabe successfully realize the LPL with a duration longer than 1 hour in a purely organic system, which can be even comparable to some excellent inorganic materials. However, partially for the unclear structure‐property relationship, organic LPL materials are still rather scarce, especially for the stable ones in air or aqueous solution. In this review, we present the recent progress in organic LPL, mainly focusing on the material design strategy and internal mechanism. It is anticipated that the deep understanding can be beneficial for the further development of organic LPL materials with good stability in air and even aqueous phase.

## Graphical abstract

This review introduces the recent progress of organic long persistent luminescence lasting for minutes or even hours. Specifically, the material design strategy and internal mechanism behind them are mainly discussed, with the aim to guide the further development of practical organic LPL materials.

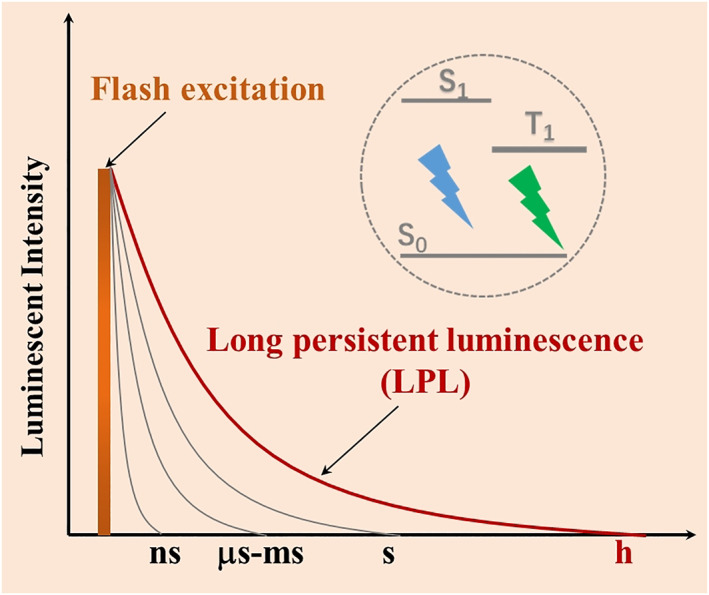

## 1 INTRODUCTION

Long persistent luminescence (LPL) is the phenomenon in which a substance glows in the dark for minutes, hours, or even days after being excited.^[1]^ Legendary luminous pearl is a typical example of natural LPL materials, which frequently appeared in some mythological stories of ancient for its unique luminescent behavior. Modern scientific research has revealed its mystery and showed that the legendary luminous pearl is a kind of fluorite mineral containing rare earth elements. Upon light irradiation, the excitation energy will be stored in excited states and then slowly released as LPL. Benefiting from the deep understanding of luminescent mechanism in the legendary luminous pearl, many artificial LPL materials with excellent performance have been successfully developed and applied in daily life, such as in architectural decorations, safety signs, watch dials, and glow‐in‐the‐dark toys.^[2]^ Also, because of the long emission lifetime, they are being explored for the application of in vivo time‐resolved bio‐imaging.^[3]^

At present, most LPL materials are based on inorganic systems, whose afterglow could even last for more than 10 hours.^[4]^ Regardless of their excellent performance, they still suffered from a series of problems. Taking the most commercial glow‐in‐the‐dark paints as examples, they usually use SrAl_2_O_4_ doped with rare elements (i.e., Eu and Dy), and the corresponding fabrication temperatures need higher than 1000°C.^[5]^ Furthermore, the existence of metal elements in inorganic systems may lead to the potential toxicity, which will be not conductive to their practical applications. If the purely organic LPL materials are developed, the advantages of low cost, easy processing and good biocompatibility can be expected.^[6]^

**FIGURE 1:**
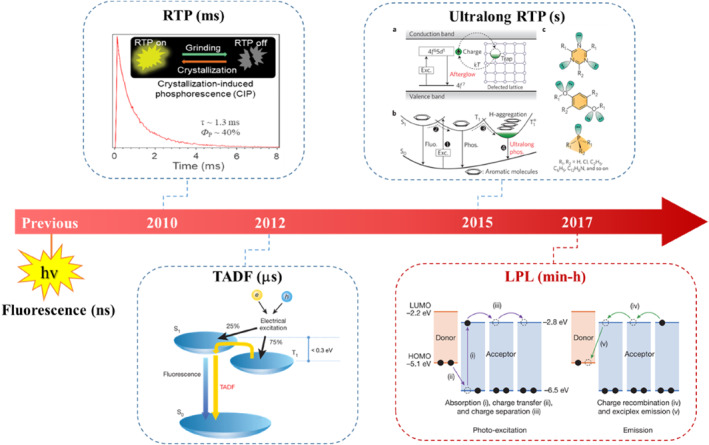
Modern research of organic luminescent materials. (1) Room temperature phosphorescence (RTP): Tang and co‐workers reported a series of organic luminogens with emission lifetimes in millisecond (ms) for the crystallization‐induced phosphorescence effect in 2010. (2) Thermally‐activated delayed fluorescence (TADF): Adachi and co‐workers developed a class of efficient TADF materials with emission lifetimes in microsecond (μs) and successfully realized high electroluminescence efficiency based on them in 2012. (3) Ultralong RTP: Huang and co‐workers successfully achieved ultralong RTP emission with lifetime in second (s) based on H‐aggregation in 2015. (4) Long persistent luminescence (LPL): Adachi and Kabe demonstrated the organic LPL with afterglow lasting for more than 1 hour (h) in 2017. Adapted with permission.^[9]^ Copyright 2010, American Chemical Society. Adapted with permission.^[10]^ Copyright 2012, Springer Nature. Adapted with permission.^[11]^ Copyright 2015, Springer Nature. Adapted with permission.^[12]^ Copyright 2017, Springer Nature.

For purely organic systems, the spin‐orbit coupling is thought to be weak and the corresponding intersystem crossing (ISC) between singlet and triplet state is usually hard.^[7]^ Therefore, when photons are absorbed by organic molecules to form singlet excitons, they will decay as fluorescence with nanosecond lifetime directly. To prolong the emission lifetime of organic systems, their excited state process should be adjusted. In recent years, related research has become a hot topic and great processes have been made (Figure 1).^[8]^ For example, in 2010, Tang and co‐workers observed the unique room temperature (RT) phosphorescence (RTP) with millisecond lifetime from the crystals of benzophenone and its derivatives.^[9]^ In these crystals, the efficient intermolecular interactions could be formed, thus restricting the non‐radiative transition and promoting the occurrence of RTP emission. Correspondingly, they termed this phenomenon as “crystallization‐induced phosphorescence (CIP)”. Besides, Adachi and coworkers reported a series of efficient organic luminogens with thermally activated delayed fluorescence (TADF) effect in 2012.^[10]^ In these compounds, the twisted D‐A structure could minimize the energy gap between S_1_ and T_1_ states, leading to the efficient ISC and reverse ISC transition between them. Accordingly, the TADF emission with microsecond lifetimes could be detected. Nevertheless, no afterglow could be observed in these organic RTP and TADF systems. Until 2015, Huang and coworkers proposed H‐aggregation to stabilize the triplet state, and ultra‐long RTP emissions lasting for several seconds were successfully realized.^[11]^ In comparison with the inorganic LPL materials, the afterglow time needs to be further prolonged.

The breakthrough of purely organic LPL materials was achieved in 2017, in which Adachi and Kabe demonstrated persistent luminescence lasting for longer than 1 hour at RT based on a blend of organic donor and acceptor molecules.^[12]^ Until then, it was certain that organic LPL systems could achieve the similar performance as inorganic ones through rational design. Thanks to the efforts of scientists, the organic LPL systems have been greatly enriched based on diverse mechanisms in recent years.^[13]^ In turn, the internal mechanisms have largely determined the properties of LPL materials, including the required excitation power and water/oxygen stability, which are of great importance for practical applications. In this short review, we would like to present the recent advances in organic LPL, mainly focusing on the material design strategy and internal mechanism, with the aim to guide the further design of practical organic LPL materials.

## 2 LPL SYSTEMS

### 2.1 Amorphous LPL systems induced by charge transfer

As early as 1997, Yamamoto and coworkers successfully realized the LPL lasting for longer than 10 hours at 20 K.^[14]^ In their systems, compound TMB with two‐photon ionization potential acted as the emitting guest, while rigid polymer matrixes were utilized to stabilize the radical cations of the guests. Upon excitation, electrons from the photo‐ionized TMB accumulated in the polymer matrixes and slowly recombined with the radical cations to generate LPL. However, this two‐photon ionization process required an intense excitation source and very low temperatures, not conductive to the development of low‐power excited LPL at RT.

**FIGURE 2:**
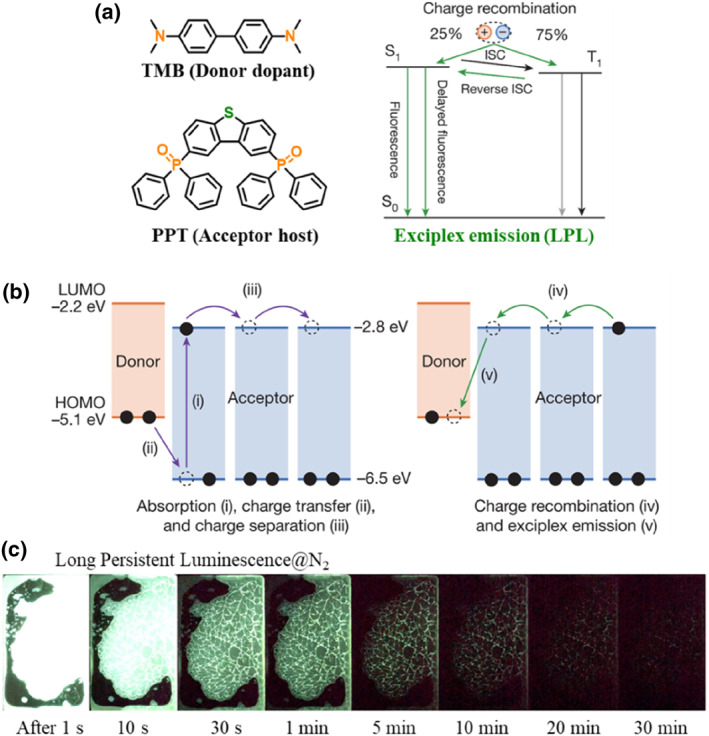
(a) Organic LPL system with exciplex emission; (b) Internal mechanism of LPL; (c) LPL photos under nitrogen atmosphere. Adapted with permission.^[12]^ Copyright 2017, Springer Nature.

To overcome the disadvantages of the two‐photon ionization process, Adachi and Kabe constructed a host‐guest doping system based on organic donor and acceptor molecules to demonstrate the low‐power excitation of OLPL at RT.^[12]^ As shown in Figure 2, electron donor of TMB still acted as dopant, while acceptor of PPT was selected as host to provide a rigid amorphous environment. Upon low‐power excitation, the charge‐transfer states could be formed between TMB donor and PPT acceptor with matched energy levels. Then, the generated PPT radical anions diffused by the hopping of charges among the PPT molecules, leading to the long‐lived charge separated states. With the restricted non‐radiative transition in a rigid environment, long‐lasting exciplex emission could occur at RT through gradual recombination of the PPT radical anions and TMB radical cations. Therefore, after a low‐power excitation of 500 μW, more than 1 h of LPL was achieved for this organic system. It was a pity that the LPL emission could not be realized in air since the photo‐generated radical anions and cations were unstable in the presence of oxygen.^[13]^

**FIGURE 3:**
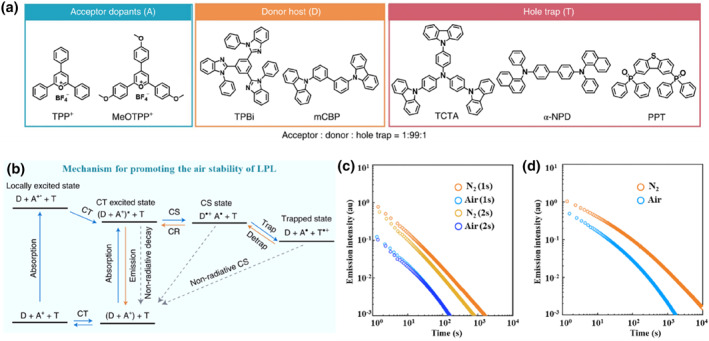
(a) The ternary LPL systems with promoted air stability; (b) Schematic diagram of a p‐type LPL system with a hole‐trapping mechanism; (c) LPL duration and cycle performance under nitrogen and air of TPP^+^/TPBi film; (d) LPL duration under nitrogen and air of TPP^+^/TPBi/TCTA film excited at 365 nm. Adapted with permission.^[15]^ Copyright 2022, Springer Nature.

Generally, the oxygen‐quenching effect in OLPL systems was mainly carried out in two aspects, namely photoreaction with and energy transfer to oxygen. To promote the air stability of organic LPL induced by charge transfer, Adachi and co‐workers constructed a series of ternary doping systems with acceptor dopant, donor host and hole trap (Figure 3).^[15]^ In comparison with the above work, two main changes have been made in these systems: (1) electron‐donor was selected as host to support carrier diffusion, which could largely restrict the quenching of oxygen through photoreaction; (2) additional hole‐trapping materials were introduced to further stabilize the charge‐separated state. Similarly, upon low‐power excitation, the charge transfer and separation between donor and acceptor could occur. Then, the generated radical‐cations (holes) diffused among the electron donor molecules with high concentration to form a long‐lived charge‐separated state. During this process, the oxygen with a reduction potential of −3.5 eV was hard to quench the diffused holes through photoreaction. If the additional hole‐trapping material was introduced, the charge‐separated state could be further stabilized for the formation of the trapped state with lower energy. Therefore, the LPL duration time was considerably prolonged from TPP^+^/TPBi (1830 s) to TPP^+^/TPBi/TCTA film (14600 s) under nitrogen condition. In air, these films could also exhibit LPL, but the corresponding durations would be shortened to 1685 s (Figure 3c,d). This was because the energy transfer from the triplet excitons to molecular oxygen could not be prevented perfectly in amorphous films, which was one of the main difficulties in the development of air‐stable LPL systems.

### 2.2 Amorphous LPL systems induced by two‐photon ionization

Besides charge transfer, the two‐photon ionization mechanism has also been utilized in the realization of LPL at RT. In 2020, Samuel and co‐workers designed and synthesized a TADF luminogen of CzPhAP. In the measurement of its emission lifetime in doped films, it was found that the slopes of the emission decay curves varied with different numbers of excitation shots.^[16]^ The more the excitation shots, the longer the emission lifetime. With 10,000 excitation shots, all the CzPhAP:PPT/TPBi/PMMA films showed emission lasting up to 1 s, which was much different from other TADF molecules. To explore the possible LPL property, the thick CzPhAP:PPT/TPBi/PMMA films were fabricated with CzPhAP as the emitting guest. Taking CzPhAP:PMMA film as an example, after 365 nm LED irradiation for 1000 s to occur two‐photon ionization, obvious LPL emission lasting over 400 s could be observed under vacuum at RT. Similar phenomena were observed in CzPhAP:PPT/TPBi films. In comparison with the LPL materials induced by charge transfer between donor and acceptor, the selection of the host materials was wider for the systems based on two‐photon ionization mechanism. Particularly, the cheap polymer PMMA could also be employed as the host, which opened the door to develop cost‐effective LPL materials with good processability. In 2024, Liang and coworkers even realized the sunlight‐activated LPL emission with a transparent and flexible polymer of poly(ethylene glycol terephthalate) (PET) as host and carbazole derivative as the guest, further enhancing their practical value.^[17]^

**FIGURE 4:**
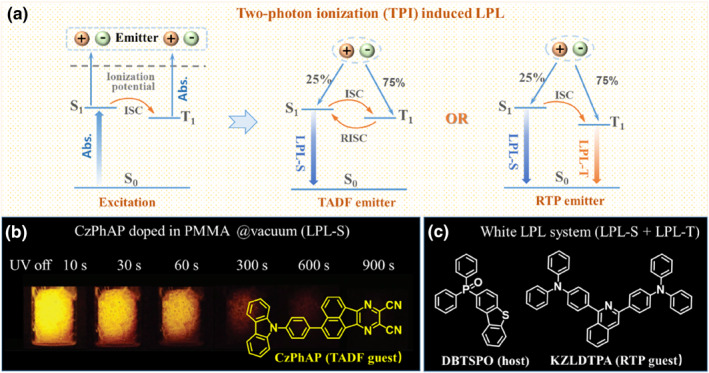
(a) Schematic diagram of two‐photon ionization induced LPL with TADF or RTP emitter; (b) LPL system based on TADF guest. Adapted with permission.^[16]^ Copyright 2020 WILEY‐VCH Verlag GmbH & Co. KGaA, Weinheim; (c) White LPL system based on RTP guest.

Further on, Zheng and co‐workers successfully realized the white LPL emission with the introduction of RTP guest (Figure 4c).^[18]^ According to the excited state process of two‐photon ionization induced LPL, the recombination of photo‐induced electron and hole would lead to the formation of 25% singlet excitons and 75% triplet excitons. If the emitting guest possessed the ability of fluorescence‐phosphorescence dual emissions, their complementary color would lead to the white LPL emission. In their work, compound DBTSPO with excellent capability to stabilize the ionic radicals was selected as host, while series of triphenylamine derivatives with phosphorescence emission potential were chosen as dopants. After two‐photon ionization, LPL with duration ranging from 20 min to around 40 min could be detected for these systems. Meanwhile, the afterglow profiles of these materials exhibited excellent consistency throughout the entire afterglow process, whose color could be rationally tuned from cyan (0.19, 0.22), cold white (0.31, 0.35), standard white (0.33, 0.33) to warm white (0.31, 0.46), according to the different fluorescence‐phosphorescence ratios from dopant molecules.

### 2.3 Amorphous LPL systems induced by charge trapping

**FIGURE 5:**
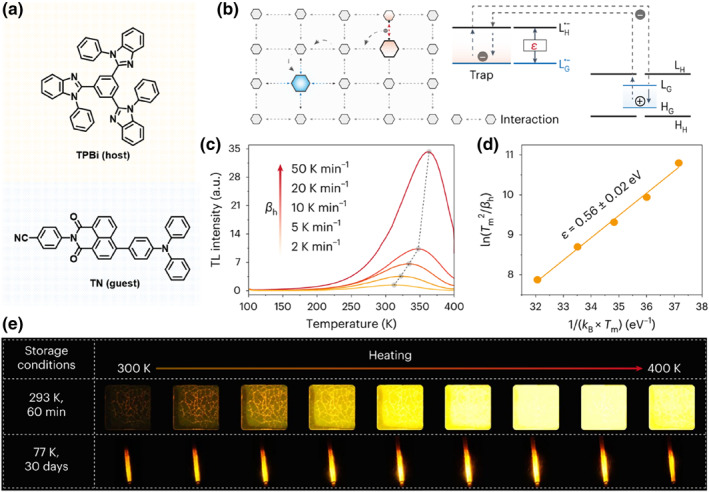
(a) Organic LPL system based on TPBi and TN; (b) Emission mechanism of trap‐induced LPL in a host–guest doping system; (c) Thermoluminescence (TL) glow curves with different heating rates of 2, 5, 10, 20 and 50 K min^−1^. The phosphor was first excited by UV light for 5 min at 100 K; (d) Estimation of trap depth with the Hoogenstraaten method; (e) Photographs of the thermoluminescence at temperatures from 300 to 400 K (Notation: after excitation with a UV lamp, the phosphor was immediately stored at room temperature for 1 h and at 77 K for 1 month in the dark). Adapted with permission.^[19]^ Copyright 2024, Springer Nature.

For inorganic LPL materials, the charge trap among the lattice has been demonstrated to play a significant role and the corresponding persistent luminescence could be well regulated through controlling the type of trap. Inspired by it, Xie and co‐workers proposed the strategy to introduce charge trap states in organic phosphors, thus realizing controllable persistent luminescence by storing and releasing photon energy.^[19]^ As shown in Figure 5, they selected TPBi with fast electron mobility as host and TN with TADF effect as guest to construct an amorphous doping system through melt‐quenching method. After continuous 365 nm UV irradiation for 5 min, orange LPL lasting for more than 24 could be detected by a photomultiplier tube (PMT) under oxygen‐free conditions. Because of the existence of charge trap for this system, typical thermoluminescence (TL) effect could be observed. Correspondingly, after natural decay for 10 min at RT (293 K), the emission intensity of the TN@TPBi film was found to show a rapid increase by 1.52, 35.2 and 63.3 times as the temperature was raised to 300, 350 and 400 K, respectively. After careful measurements of the TL spectra under different rates of temperature increase, the trap depth (*ε*) of the TN@TPBi film was estimated to be 0.56 ± 0.02 eV, which corresponded well to the energy gap between LUMOs of TPBi^•−^ and TN^•−^. Based on this, it was thought that the traps in the TN@TPBi film stemmed from the activation energy required to escape from the LUMO of TN^•−^ (that is, TN^•−^^*^) to the LUMO of TPBi^•−^ (TPBi^•−^^*^). Further on, through regulating the LUMOs of host^•−^ and guest^•−^ that heavily related to the depth of traps, the emissions of LPL and TL for the newly developed doping systems were controllably tuned from blue (507 nm) to red (669 nm), while the afterglow time lasted for 0.86 h or more than 12 h at RT. During the same period, Li and coworkers have also reported the trap‐induced LPL behavior of small molecule crystals, in which the hour‐level radioluminescence was successfully merged.^[20]^ Based on these works, the significant role of the charge trap in organic LPL could be well certified.

### 2.4 Air‐stable LPL systems in crystal state

**FIGURE 6:**
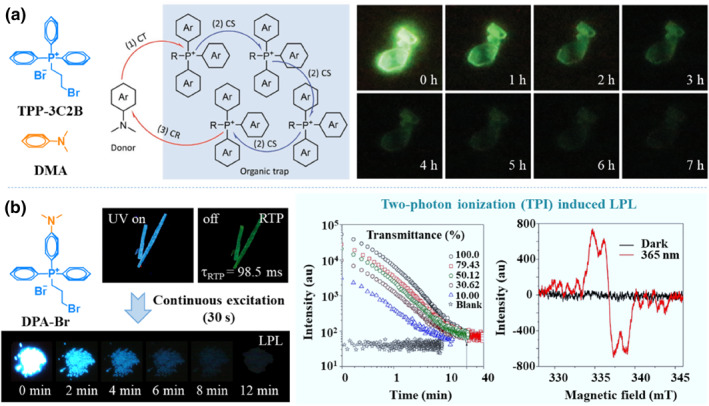
(a) Bi‐component air stable LPL crystal with charge transfer. Adapted with permission.^[21]^ Copyright 2020, WILEY‐VCH Verlag GmbH & Co. KGaA, Weinheim; (b) Single‐component air stable LPL crystal with two‐photon ionization. Adapted with permission.^[22]^ Copyright 2022, American Chemical Society.

In comparison with the amorphous state, crystals usually present a more rigid environment, and can easily restrict the diffusion of molecular oxygen, which will be much helpful to realize the air‐stable LPL. In 2020, Tang and co‐workers utilized the phosphonium salt of TPP‐3C2B as an electron acceptor/host and neutral DMA as a donor/guest to construct an LPL system with charge transfer (Figure 6a).^[21]^ When they were mixed together with optimized molar ratios to form co‐crystals, green LPL lasting for about 7 h could be achieved in air. It was believed that the crystalline nature of TPP‐3C2B:DMA protected the photo‐generated radicals from atmospheric oxygen and suppressed the nonradiative deactivation efficiently. Besides, the phenyl rings decorating the phosphonium core of TPP‐3C2B stabilized the radical via steric protection to hinder unwanted reactions, while its ionic nature aided in the crystallization. These factors jointly contributed to the realization of air‐stable LPL emission. Furthermore, these LPL crystals were found to exhibit excellent stability, showing no visible changes to its afterglow duration even after being kept for more than 45 days under ambient dark conditions.

To simplify the LPL system, Tang and coworkers designed and synthesized a new phosphonium salt of DPA‐Br by combining a donor of *N,N*‐dimethylaniline moiety and an acceptor of arylphosphonium in a single molecular system (Figure 6b).^[22]^ In crystal state, it showed blue fluorescence and green RTP afterglow upon 365 nm UV irradiation with a short exposure time. Its RTP lifetime was measured to be just 98.5 ms. If the excitation of 365 nm UV light was prolonged to about 30 s, the DPA‐Br crystals could exhibit persistent blue afterglow emission that lasted up to 12 min under ambient conditions, showing the typically air‐stable LPL characteristic. Since the emission color of LPL was consistent with its fluorescence emission, the LPL should decay from the excited singlet state. Furthermore, its LPL property was found to be heavily related to the excitation power. The peak intensities were decreased and emission durations were shortened gradually by using neutral density filters to reduce excitation transmittance, thereby decreasing the excitation power intensity. Thus, it was thought that the two‐photon ionization should be the main origin for its LPL effect, which was different from the binary LPL system mentioned in Figure 6a. Nevertheless, the charge‐transfer induced exciton dissociation and resultant LPL was hard to be excluded for the existence of electron‐donor and acceptor groups in compound DPA‐Br.

### 2.5 Stable LPL system in aqueous solution

In addition to air stability, the development of stable LPL materials in aqueous solution is also of great importance for their applications in time‐resolved bio‐imaging. Under light‐irradiation, the biological tissues could also exhibit fluorescence, which would largely decrease the signal‐to‐background ratio (SBR) of bio‐imaging for luminescent probes. Because of the ultra‐long emission lifetime of LPL materials, the signal of the bio‐imaging could be acquired after stopping the light irradiation, thus eliminating the interference of background fluorescence and improving the imaging quality.

**FIGURE 7:**
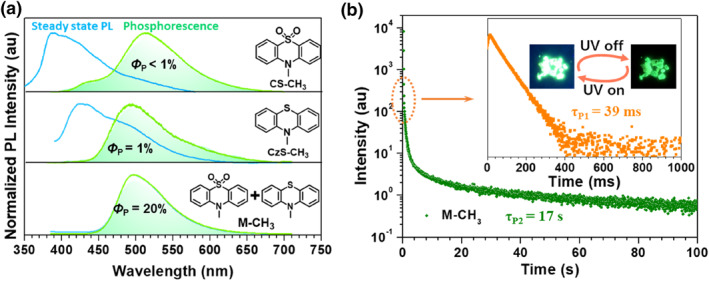
(a) Normalized steady‐state PL and phosphorescence spectra of CS‐CH_3_, CzS‐CH_3_ and M‐CH_3_ crystals; (b) Phosphorescence decay curves of M‐CH_3_ crystal. Adapted with permission.^[23]^ Copyright 2021, WILEY‐VCH Verlag GmbH & Co. KGaA, Weinheim.

As early as 2018, Li and co‐workers successfully demonstrated the great advantages of time‐resolved bio‐imaging based on RTP materials.^[8b]^ In 2021, they successfully developed a series of stable LPL materials in aqueous solution and systematically studied their potential in bio‐imaging.^[23]^ In their work, two phenothiazine derivatives (CzS‐CH_3_ and CzS‐C_2_H_5_) were selected as guests, and their corresponding dioxide derivatives (CS‐CH_3_ and CS‐C_2_H_5_) were selected as hosts to yield the host‐guest doping systems of M‐CH_3_ and M‐C_2_H_5_, respectively, since they were found to show moderate RTP effects and matched energy levels in previous works.^[24]^ When they were mixed together with a mass ratio of 1:100 to co‐crystallize, the resultant co‐crystals showed boosted RTP efficiency and much prolonged RTP lifetime. Taking M‐CH_3_ as an example, a high RTP efficiency of 20% was achieved for it, while the individual CzS‐CH_3_ or CS‐CH_3_ crystal gave a low phosphorescence efficiency of no more than 1% (Figure 7). Besides, two components were found for its RTP emission. Among them, one component was bright and relatively short (39 ms), while the other was weak and could last for tens of minutes, with a lifetime of 17 s. Careful studies indicated that the former was from the triplet exciplex based on CzS‐CH_3_ and CS‐CH_3_, since the formation of it could promote the ISC transition between the singlet and triplet states. Besides, the triplet exciplex was found to show a close T_1_ state with RTP host of CS‐CH_3_, which could lead to the recycling of triplet excitons between them, thus largely increasing the RTP lifetime of RTP host, from 245 ms in the single‐component state to 17 s in the host‐guest doping state.

**FIGURE 8:**
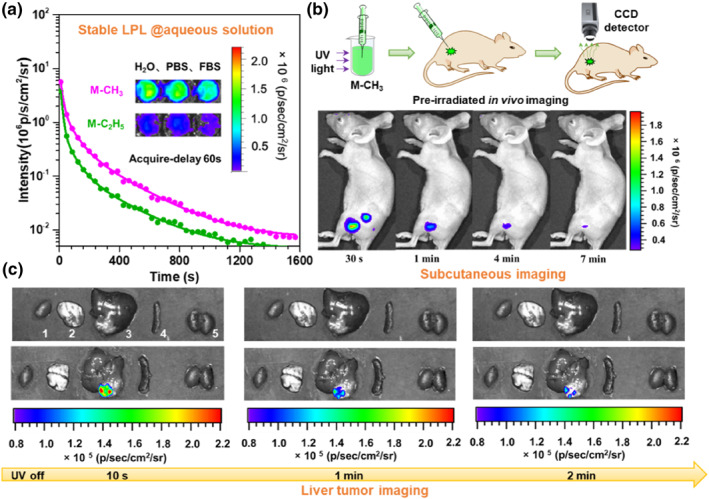
(a) LPL decay curves of M‐CH_3_/M‐C_2_H_5_ nanocrystals in aqueous solution, acquired by an IVIS instrument in bioluminescent mode. Inset: afterglow images of nanocrystals in different aqueous media acquired at 60 s after stopping light excitation; (b) The demonstration of subcutaneous imaging of mice based on LPL materials; (c) Liver tumor imaging of mice based on LPL materials. Adapted with permission.^[23]^ Copyright 2021, WILEY‐VCH Verlag GmbH & Co. KGaA, Weinheim.

The top‐down method was employed to formulate M‐CH_3_ nanocrystals using the biocompatible amphiphilic co‐polymer PEG‐b‐PPG‐b‐PEG (F127) as the encapsulation matrix, and a long afterglow up to 25 min could still be observed in its aqueous solution (Figure 8). After confirming the rather low cytotoxicity and in vivo side toxicity of M‐CH_3_ nanocrystals, the applications of pre‐irradiated subcutaneous imaging and liver tumor imaging were explored. In pre‐irradiated subcutaneous imaging, phosphate buffered saline solution of M‐CH_3_ nanocrystals was first irradiated by a 365 nm UV lamp for 30 s, and then subcutaneously injected into mice, followed by imaging. The afterglow signals could be detected for longer than 7 min, and the signal‐to‐background ratio (SBR) of subcutaneous imaging at 30 s was as high as 310, demonstrating the great advantage of LPL materials in time‐resolved bio‐imaging with negligible background interference.^[25]^ Further on, this LPL material was found to show excellent performance in cancer diagnosis. As shown in Figure 8c, the liver tumor of mice could be clearly labeled by M‐CH_3_ nanocrystals and a high SBR value of 158 was obtained, which represented one of the highest values among reported optical imaging studies of orthotopic liver tumors.^[26]^ However, it is a pity that the tumor imaging could just be realized in ex vivo experiment for the inferior tissue penetration of green emission from M‐CH_3_. For the further applications of in vivo imaging, the LPL materials with long wavelength emission need to be developed.^[27]^

In comparison with amorphous or crystal state, the LPL materials in aqueous solution are much less and more difficult to be obtained, especially for those that can be applied in bio‐imaging. In the aqueous phase, the LPL emitters need to be dispersed as single molecules or nanoaggregates, which will make them to be easily quenched by molecular oxygen in solution. Also, the water molecules could interact with the LPL emitters to accelerate their non‐radiative motion. If they are utilized in bio‐imaging, the complex physiological environment will lead to more quenching possibilities. Thus, more intelligent material design strategies are needed for the development of stable LPL materials in aqueous solution to meet the requirements of biological applications.

## 3 CONCLUSIONS AND OUTLOOK

In this review, five kinds of organic LPL materials were presented with selected examples, including amorphous LPL systems induced by charge transfer, amorphous LPL systems induced by two‐photon ionization, amorphous LPL systems induced by charge trapping, air‐stable LPL systems in crystal state and stable LPL system in aqueous solution. For the amorphous systems induced by charge transfer, two‐photon ionization and charge trapping, they were mainly fabricated through the melt‐casting method. This method showed the advantages of fast and easy preparation, while it also put forward higher requirements for the thermal stability of materials. In comparison with two‐photon ionization, the photo‐induced charge transfer between electron donor and acceptor usually occurred in a much lower power excitation. However, careful matching between electron donor and acceptor was required for the LPL induced by charge transfer. Besides, these three kinds of LPL materials were both difficult to realize air stabilization, since the energy transfer from the triplet excitons to molecular oxygen could not be prevented perfectly in amorphous films. Therefore, crystallization might be a more viable way to develop air‐stable LPL systems.

At present, several LPL systems in the crystal state have been reported based on phosphonium salts, which were found to show much better air stability than those in the amorphous state. However, the construction of these materials had a certain degree of chance, since the minor change in alkyl chain or counter ion for phosphonium salts would both lead to the disappearance of LPL property. These results indicated that the internal mechanisms for these LPL crystals were more complicated, and the aggregation structure, including molecular packing mode and intermolecular interaction, should be considered more seriously.

For practical applications, the LPL systems with both air stability and water stability are urgently needed and would be the main focus in the further research. One strategy, though, has been proposed to physically encapsulate nanocrystals in surfactants to prepare stable LPL materials in aqueous solution, but it is still not enough. If the method of chemical bonding could be developed, the long‐term stability and environmental stability of LPL materials in the aqueous phase could be further increased. Also, the emission colors of the corresponding LPL materials were limited in the green region, which would largely limit their practical applications, especially for the in vivo bio‐imaging of deep tissues. To achieve them, there is still a long way to go.

Last but not least, the concept of organic LPL is still not so clear now, and some researchers confuse it with ultralong RTP effect. During the RTP process, it will undergo the photoexcitation, then ISC transition from singlet to triplet state, and finally radiative decay from triplet state.^[28]^ This procedure usually results in an afterglow of less than 1 minute. For LPL emission process, it could be roughly divided into four steps in most cases: (1) excitation of molecules; (2) formation of charged carriers; (3) storage and release of charged carriers; (4) recombination of charged carriers.^[29]^ Step 3 will give the corresponding system ultralong emission lifetime, so most of the LPL can last for minutes, hours, or even days after being excited. In step 4, the recombination of charged carriers would populate the singlet and triplet excitons at the ratio of 1:3 according to the spin statistics, although this has not been well demonstrated by experimental results. Therefore, the LPL emission could contain both fluorescence and phosphorescence, and integrating the RTP or TADF property into the LPL systems would increase the resultant exciton utilization and emission efficiency. Nevertheless, it could not be excluded that the afterglow of RTP or even TADF could be prolonged to minutes or even hours without undergoing these four steps. At that time, they should also be attributed to LPL.

## CONFLICT OF INTEREST STATEMENT

The authors declare no conflicts of interest.

## Data Availability

No data are available, thanks.
